# Pathogenesis of acute encephalopathy in acute hepatic porphyria

**DOI:** 10.1007/s00415-023-11586-5

**Published:** 2023-02-09

**Authors:** Elena Pischik, Katrin Baumann, Alla Karpenko, Raili Kauppinen

**Affiliations:** 1 Department of Neurology, Consultative and Diagnostic Center with Polyclinics, St. Petersburg, Russia; 2 Department of Medicine, University Central Hospital of Helsinki, Helsinki, Finland; 3 Department of Gynecology and Obstetrics, University Central Hospital of Helsinki, Helsinki, Finland; 4 Department of Radiology, Consultative and Diagnostic Center with Polyclinics, St. Petersburg, Russia; 5 High Technology Institution, North-Western State Medical University, St. Petersburg, Russia; 6 Biomedicum-Helsinki2, Tukholmankatu 8C, 00029 HUS Helsinki, Finland

**Keywords:** Porphyria, Encephalopathy, Seizures, MRI, PRES, Vasoconstriction, Cerebral venous sinus thrombosis, PEPT2

## Abstract

Acute encephalopathy (AE) can be a manifestation of an acute porphyric attack. Clinical data were studied in 32 patients during AE with or without polyneuropathy (PNP) together with 12 subjects with PNP but no AE, and 17 with dysautonomia solely. Brain neuroimaging was done in 20 attacks during AE, and PEPT2 polymorphisms were studied in 56 subjects, 24 with AE. AE manifested as a triad of seizures, confusion and/or blurred vision. Symptoms lasting 1–5 days manifested 3–19 days from the onset of an attack. 55% of these patients had acute PNP independent of AE. Posterior reversible encephalopathy syndrome (PRES) was detected in 42% of the attacks. These patients were severely affected and hyponatremic (88%). Reversible segmental vasoconstriction was rare. There was no statistical difference in hypertension or urinary excretion of porphyrin precursors among the patients with or without AE. In 94% of the attacks with AE, liver transaminases were elevated significantly (1.5 to fivefold, *P* = 0.034) compared to a normal level in 87% of the attacks with dysautonomia, or in 25% of patients with PNP solely. PEPT2*2/2 haplotype was less common among patients with AE than without (8.3% vs. 25.8%, *P* = 0.159) and in patients with PNP than without (9.5% vs. 22.9%, *P* = 0.207), suggesting a minor role, if any, in acute neurotoxicity. In contrast, PEPT2*2/2 haplotype was commoner among patients with chronic kidney disease (*P* = 0.192). Acute endothelial dysfunction in porphyric encephalopathy could be explained by a combination of abrupt hypertension, SIADH, and acute metabolic and inflammatory factors of hepatic origin.

## Introduction

Acute encephalopathy can be a manifestation of a protracted or severe attack of acute hepatic porphyria (AHP). An acute attack manifests typically with acute abdominal pain and signs of autonomic dysfunction, peripheral neuropathy (PNP) and central nervous system (CNS) involvement, accompanied by excess of plasma and urine porphyrins and precursors [1]. Accumulation of aminolevulinic acid (ALA), and porphobilinogen (PBG) in the circulation is initialised by triggering factors, such as certain medications, fasting, hormonal changes during the luteal phase of the menstrual cycle, and alcohol, via up-regulation of ALA synthase (ALAS1). ALAS1 is the rate-limiting enzyme of the haem biosynthesis in the liver. Each of the four AHPs (acute intermittent porphyria, AIP, hereditary coproporphyria, HCP, variegate porphyria, VP, and ALA dehydratase, ALAD, deficiency porphyria) results from a partial deficiency in one of the enzymes in the haem biosynthesis. All of them manifest with a similar clinical picture of an attack.

Mild mental symptoms such as anxiety, insomnia, irritability and even mild cognitive decline occur at the preliminary stage of an attack, independent on its severity, commonly prior to abdominal pain [1]. Acute encephalopathy manifests as aberrant behaviour, hallucinations, confusion, or decreased consciousness (19–58% of attacks), hyponatremia (25–61%) or seizures (1–20%) with or without hyponatremia (Table 1). Seizures are transient, presenting only in severe attacks with AE, and do not occur in remission [1].

**Table 1:** Clinical manifestations during an acute attack in different patient series

Signs and symptoms	Unselected patients with AHP						Selected AHP patients with neuropathy or encephalopathy		
Series Number of attacks/patients	(1) *n* = 252	(2) *n* = 50	(3) *n* = 40	(4) *n* = 88	(5)^a^ *n* = 51/22	(6) *n* = 112/24	(7) *n* = 25	(8) *n* = 12	(9)^b^ *n* = 46
I. Autonomic dysfunction (%)									
Abdominal pain	85	94	95	95	96	97	100	83	83
Tachycardia (> 80 per min)	28	64	80	85	79	38	96	92	55
Hypertension	40	54	36	55	57	74	52	67	70
Constipation	48	84	48	80	78	27	94	75	28
Vomiting and nausea	59	88	43	80	84	79	44	42	32
Bladder paresis	n/a	n/a	12	n/a	n/a	n/a	54	33	n/a
II. Peripheral neuropathy (%)									
Pain in the back and limbs	n/a	52	50	70	25	n/a	68	75	n/a
Paresis/muscle weakness	42	68	60	50	8	10/46^c^	100	83	53
Low/absent tendon reflexes	n/a	54	29	n/a	n/a	n/a	97	83	63^d^
Respiratory paresis	n/a	10	9	20	0	n/a	55	67	n/a
Cranial neuropathy	n/a	28	54	15	0	n/a	69	58	n/a
Neuropathic sensory loss	n/a	38	26	25	n/a	n/a	59	42	n/a
III. Encephalopathy (%)									
Mental symptoms	55	58	40	40	19	1^e^	86	92	72
Seizures	10	16	20	20	1	5	21	33	85
Coma	n/a	n/a	10	n/a	0	n/a	n/a	25	10^d^
Headache	n/a	n/a	5	0	0	n/a	0	33	13
Blurred vision	n/a	6	6	n/a	2	n/a	7	8	38
Babinski signs	n/a	10	3	n/a	n/a	n/a	3	50	n/a
IV. Metabolic changes (%)									
Hyponatremia	n/a	25	26	61	32	31	28	42	55
Transaminases increased	n/a	n/a	13	n/a	n/a	n/a	69	100	62^d^
Pink/red/dark urine	n/a	n/a	74	90	90	n/a	100	92	28

^1^Waldenström [67]; ^2^Goldberg [68]; ^3^Stein and Tchudy [69]; ^4^Mustajoki and Koskelo [70]; ^5^Mustajoki and Nordmann [71]; ^6^Hift, Meissner [72]; ^7^Ridley [11]; ^8^Pischik [73]; ^9^Jaramillo-Calle et al. [29]

*n/a*, not applicable

^a^All cases with early treatment with heme arginate

^b^Review: cases were collected from other articles with case reports of PRES

^c^% of attacks/patients

^d^Not mentioned in the review, added after our analysis from the same original articles

^e^Only psychosis is included

Focal CNS involvement is rare during an attack and may easily be neglected because of its short duration. Single cases of transient cortical blindness, cerebellar ataxia, dysphasia, anosognosia, apraxia, vertigo or dizziness, Babinski signs or central tetraparesis have been reported during an attack [2–14]. The frequency of severe encephalopathy with confusion, unconsciousness or hallucinations is difficult to evaluate since mental symptoms have been pooled in earlier series (Table 1).

ALA is the only potent neurotoxin known to be associated with the onset of acute attacks [15]. It has very low permeability through the blood–brain barrier (BBB), ~1% of the serum level demonstrated in animal studies and patients with AIP [15–17]. Thus, direct neurotoxicity of ALA in the brain unlikely explains AE [15].

Haem deficiency in the CNS has been suggested, but there is only minor evidence from the murine HMBS-/- model to support it [18]. Another study has demonstrated normal expression and activity of the haem-containing enzymes in the brain of the same mice model [19]. The findings of brain autopsies of the patients, who died of an attack, have been unspecific. They range from macroscopically normal brain with mild gliosis to diffuse loss of neurons and chromatolysis, and thus, have not elucidated the pathogenesis of AE [4, 6, 15].

The human peptide transporter two (PEPT2) is abundantly expressed on the apical membrane of the proximal tubule in the kidney and the epithelial cells of the choroid plexus [20–22]. PEPT2 mediates the reabsorption of ALA in the proximal tubule, and efflux in the cerebrospinal fluid (CSF), limiting the exposure to ALA [23–25]. Previous haplotype analysis has revealed two main PEPT2 variants (PEPT2*1 and PEPT2*2), which are present in substantial frequencies in all ethnic groups and may have an impact on the phenotype of AHP patients [26].

PEPT2*1/1 genotype, with higher ALA reabsorption affinity in the proximal tubular cells has been associated with lower renal function than low affinity variants (PEPT2*1/2 and PEPT2*2/2), whereas PEPT2*2/2 haplotype may increase brain ALA levels [27, 28]. We investigated the distribution PEPT2 haplotypes in our cohort and analysed whether the PEPT2 variants impact porphyria-induced encephalopathy or renal dysfunction.

During the era of neuroimaging, an increasing number of cases have been associated with posterior reversible encephalopathy syndrome (PRES) and severe AE due to AHP [29]. In this study, we have analysed the spectrum and occurrence of abnormal neuroimaging findings in 19 AHP patients with AE.

## Materials and methods

During 1996–2021, 19 well-characterised patients with AHP (total 20 attacks) had severe AE studied by brain CT or MRI. The diagnosis of an attack was detected clinically by typical symptoms such as abdominal pain and/or dysautonomia in combination with at least a fivefold increase in urinary excretion of PBG (expressed as μmol/L) compared to the upper normal limit [30]. AE was attributed to AHP only if it manifested during an attack (Table 2). Mild mental symptoms such as anxiety, irritability or insomnia were not solely sufficient for the diagnosis of AE. Clinical and biochemical data was included from additional 42 attacks with (*n* = 13) or without (*n* = 29) AE but no neuroimaging.

**Table 2:** Demographic, clinical and laboratory data on 19 patients during 20 acute attacks with encephalopathy

Demographic data			Measurement of porphyrin precursors during an acute attack and in remission					Duration (d) of pain and dysautonomia before the onset of other neurological manifestations		Routine laboratory findings		
Pt	Age	Type of AHP	U-PBG, < 9 μmol/L		U-ALA, < 34 μmol /L		Severe abdominal pain	Encephalopathy	Peripheral neuropathy	S-Na, mmol/L > 135	S-ALT, U/L < 45^c^	S-Creatinine, μmol /L < 110
			Attack	Remission	Attack	Remission						
1	26	HCP	+ + +	5^a^	n.d	41	+	5, 9 and 14	10	130	29–131	Normal
2	24	AIP	470^a^	250	310 ^a^	130	+	7	No	129	78–97	Normal
3	36	AIP	+ + +	293	−	124	+	12 and 16	11	137	17–269	122
4	35	AIP	588	147	344	60	+	8 and 14	12	119	132	Normal
5	20	AIP	609	129	291	89	+	10 and 15	No	115	128–317	193
6	22	AIP	+ + +	117	−	35	+	9 and 22	No	121	48–110	Normal
7	31	AIP	+ + +	228	340	94	+	7 and 22	7	121	23–210	Normal
8	42	AIP	+ + +	211	−	90	+	16	7	125	38	127
9	36	AIP	900^a^	300	570^a^	150	+	10	No	108	127–233	Normal
10^1b^	26	AIP	480	135	306	124	Moderate	10	No	112	115	Normal
10^2^	28		622	371	232	165	+	3 and 8	No	125	50	Normal
11	31	AIP	414	121	110	27	+	9 and 16	25	120	74	Normal
12	40	VP	840^a^	28	650^a^	−	+	4	No	130	N.D	Normal
13	26	AIP	+ + +	134	204	−	+	5	13	115	104–109	Normal
14	23	AIP	+ + +	258	−	160	Mild–moderate	3	No	138	69–128	Normal
15	36	AIP	+ + +	died	−	died	+	17, 26, 49	30	119	65–194	Normal
16	25	AIP	+ + +	114	−	110	Mild–moderate	3	No	140	52–70	Normal
17	29	AIP	+ + +	401	−	193	+	7	16	135	103–224	152
18	30	AIP	875^a^	268	829^a^	214	+	17	15	132	103–207	Normal
19	28	AIP	305	132	88	51	+	19	18	132	56–170	136

^a^Samples were taken at the nadir of encephalopathy

^b^Patient 10 had two attacks with encephalopathy within 2 years, marked as 10^1^ and 10^2^

^c^Serum ALT by the first symptom of encephalopathy—maximal S-ALT level during the attack.  − not done

The diagnosis of AIP, VP or HCP was confirmed by quantitative biochemical analysis of porphyrins and precursors (Table 2) and genetic testing [30]. All multinational patients were either treated or supervised by the authors during the attack.

Study protocol followed principles outlined in the Declaration of Helsinki, and ethical issues were approved by the Consultative and Diagnostic Centre with Polyclinics, Saint Petersburg, Russia and Central University Hospital of Helsinki, Finland.

The PEPT2 haplotype was analysed among 44 symptomatic AHP patients (34 AIP, 9 VP, 1 HCP), eight asymptomatic mutation carriers (7 AIP, 1 HCP) and four healthy relatives. Of the symptomatic patients, 24 had a history of AE including 16 subjects of the current study. Other six patients had an attack with PNP, and 14 subjects dysautonomia solely.

DNA samples extracted from peripheral blood leucocytes were amplified by PCR and sequenced using primers detecting polymorphic sites in the PEPT2 gene, in the region of exon 13 and 15 [27].

Computed tomography (CT) or magnetic resonance imaging (MRI) of the brain was performed during acute attacks (Table 3) by standard protocol. All images were of good quality and retrospectively reviewed by the same neuroradiologist (AK).

**Table 3:** Clinical and neuroimaging data and PEPT2 phenotype on 19 patients with acute encephalopathy during 20 acute attacks

	Clinical manifestations of encephalopathy					Neuroimaging			Timing of neuroimage			PEPT2 Exon 13; Exon 15
Pt	Seizures	Severe mental symptoms	Focal CNS signs	Headache	BP	Imaging	Findings	MRA	After nadir of encephalopathy	After seizures	MRI follow-up	
1	+	Confusion, Coma	Blurred vision	−	170/95	CT MRI	PRES, Sinus thrombosis	Vasoconstriction LMCA, BA	1 d	1 d	2 y gliosis MRA: 24 d normal	C/C; C/C
2	+	Confusion	No	+	180/100	MRI	PRES	Normal	1 d	1d	10 d residual / 1.5 mo. normal	C/C; C/C
3	+	Confusion	Hemiparesis, Babinski	−	160/95	CT	PRES	N.D	< 1 d	< 1d	1.5 mo. normal	C/T; C/T
4	+	Confusion	No	−	120/80	MRI	PRES	Normal	< 1 d	< 1 d	8 d normal	T/T; T/T
5	−	Confusion,	No	+	160/100	MRI	PRES	Normal	< 1 d		6 d residual/ 1.5 mo. normal	C/C; C/C
6	−	Confusion	Blurred vision dysphasia monoparesis	−	200/100	CT MRI	Negative PRES	Normal	1 d		3 d progress 1.5 mo. mini mal sequels	C/T; C/C
7^a^	+ −	Hallucinations Confusion	No	−	172/100 109/70	MRI MRI	Negative PRES	N.D	2 d 1 d	2 d	1 y normal	C/T; C/T
8	−	Confusion	No	−	155/95	MRI	PRES/Wernicke nucleus, caudatus	N.D	1 d		N.D	C/T; C/T
9	−	Confusion	Babinski	−	140/85	MRI	No bright signal in neurohypophysis	N.D	10 d		1.5 mo. normal	C/T; C/T
10^1b^	+	Confusion	No	+	190/110	CT	Negative	N.D	1 d	1 d		C/C; C/C
10^2^	−	No	Blurred vision	−	160/100	CT	Negative	N.D	1 d			C/C; C/C
11	+	Confusion	Blurred vision	−	140/80	CT	Negative	N.D	1 d	8 d		C/C; C/C
12	−	Confusion	Blurred vision	−	196/116	CT	Negative	N.D	1 d			C/T; C/T
13	+	Confusion	No	−	160/110	MRI	Negative	N.D	< 1 d	< 1 d		N.D
14	+	Lethargy	Babinski	+	176/105	MRI	Negative	N.D	3 d	10 d		C/T; C/T
15	−	Confusion	Blurred vision	−	160/100	MRI	Negative	N.D	8 d			N.D
16	−	Confusion	Vertigo	+	160/100	MRI	Negative	N.D	10 d			C/T; C/T
17	−	Hallucinations	No	−	130/80	MRI	Negative	N.D	1 d			T/T; T/T
18	−	Confusion	No	−	125/80	MIR	Negative	N.D	1 d			C/T; C/T
19	−	Confusion Hallucinations	No	−	140/80	MRI	Negative	N.D	4 d			C/T; C/C

^a^During the attack with encephalopathy MRI was preformed twice, first was unremarkable

^b^Two attacks with encephalopathy within 2 years, marked as 10^1^ or 10^2^

## Results

### The natural course of attacks with encephalopathy

Of the 19 patients studied by neuroimaging, 89% were women (*n* = 17) with median age of 30 years (Table 2). AHP was undiagnosed before the attack except in two cases (Cases 10 and 13). All patients had clinical signs of AE during the total of 20 attacks. Nine patients had abnormal findings on neuroimaging (47%), and eight of them had PRES. Ten patients (11 attacks) had normal neuroimaging despite typical AE.

Attacks were triggered by a combination of hormonal changes during the luteal phase of the menstrual cycle, prolonged fasting, porphyrinogenic drugs and/or alcohol. Two patients were at the postpartum phase (Case 11, day 8 and Case 13, day 16).

Severe abdominal pain was commonly an early sign of an attack in combination with the signs of dysautonomia (tachycardia, constipation, nausea) and mild mental symptoms (anxiety and insomnia) (Table 2). AE manifested 3–19 days from the onset of an attack. It was associated with porphyrinogenic drugs administered on the preceding day, severe hyponatremia, hypertension and in two cases with high-dose prednisolone infusion. Symptoms of AE dissipated quickly (1–5 days) but in nine cases, reoccurred after a 4–15-day interval (Table 2). Only Case 1 had prolonged confusion and coma due to central venous sinus thrombosis (CVST).

Eleven patients had motor axonal PNP independent of AE (Table 2). The clinical presentation of PNP either preceded encephalopathy or developed 1–9 days after seizures and confusion (Table 2, Fig. 2).

### Clinical manifestations of porphyric encephalopathy

Typical porphyric encephalopathy included a triad of seizures, confusion, and blurred vision. Severe mental symptoms, such as somnolence or stupor, confusion, hallucinations, and cognitive impairment, occurred acutely. They commonly preceded seizures or focal CNS signs, such as blurred vision or hemiparesis.

Seizures were present in 45% (*n* = 9) of the attacks with AE, and in 63% (*n* = 5) of those with PRES. Transient EEG findings varied from focal epileptiform discharge related to the area of MRI lesions to general slowing associated with diffuse encephalopathy. EEG findings resolved with the clinical regression of the seizures and did not reoccur during the follow-up. None of the patients had seizures after the acute attack.

Severe headache was rare and exceeded abdominal pain in intensity in 25% of the attacks (*n* = 5) (Table 3). The most severe bursting headache was evident in Case 2 when the drug-resistant hypertensive crisis manifested after treatment of seizures with porphyrinogenic anticonvulsants. It resolved rapidly with hematin.

Blurred vision was present in 30% (*n* = 6) of the attacks, and in 22% of those with PRES. Central mono- or hemiparesis was present in 10% (*n* = 2) of the attacks, both with PRES (Table 3).

Although blood pressure during AE was commonly elevated (200–125/116–80 mmHg), no statistical correlation was seen between hypertension and encephalopathy (*P* = 0.182). Only three patients had systolic pressure exceeding 180 mmHg (Table 3). Hypertension usually had an abrupt onset.

### Laboratory findings

Urinary excretion of PBG was increased > 50-fold and ALA > tenfold when compared to the normal level, but 2–4-fold when compared to the patients’ values in remission. Of note, urinary excretion of porphyrin precursors is expressed as μmol/l not as μmol/mmol creatinine which is commonly used in other European countries. Of the four patients, whose PBG and ALA urinalysis were available at the nadir of AE, three showed a 100-fold elevation of PBG and > 20-fold for ALA. All patients had constantly high urinary excretion of PBG and ALA during the follow-up (Table 2). There was no statistically significant difference in urinary ALA (*P* = 0.722) or PBG excretion (*P* = 0.813) between patients with or without AE during an attack. Routine laboratory examinations revealed hyponatremia, mild elevation of liver transaminases and creatinine in both patient groups.

Hyponatremia due to inappropriate antidiuretic hormone syndrome (SIADH) was present in 16 of 20 attacks. SIADH developed before neurological manifestations and usually lasted two weeks. Of the eight patients with PRES, seven were hyponatremic and four were severely hyponatremic (S/P-Na < 125 mmol/L, Table 2). The mean sodium values did not differ in patients with or without PRES.

Serum ALT levels were analysed retrospectively from 42 additional attacks from our patient registries for comparison. The total of 62 attacks included 12 with PNP but no AE, 17 dysautonomia solely, and 33 complicated by AE. Serum ALT became elevated between 2nd and 27th day (mean 10 days) from the onset of an attack and became normal within 1–2 months. ALT was normal in most attacks (87%) with dysautonomia (mean 40 U/L, range 9–127 U/L, normal < 50 IU/l), and in 25% of the attacks with PNP (mean 76, range 35–198 U/L). The elevation of ALT was earlier and more pronounced (1.5–fivefold; mean 129 U/L, range 50–317 U/L, *P* = 0.034) in 94% of the patients with AE (Table 2) compared to those with PNP or autonomous dysfunction solely. Thus, hepatopathy was more common and severe in the attacks with AE.

Five patients (26%) had mildly increased creatinine levels (122–193 µmol/L, normal < 90 µmol/L) during an attack (Table 2), which normalised within a month in four of them. One patient developed mild chronic kidney disease, and another had acute kidney injury during a subsequent attack. None of the patients had proteinuria or haematuria indicating failure at the tubulointerstitial level. In Case 5 a renal biopsy was normal.

At the onset of AE, no laboratory findings were related to systemic inflammation, autoimmune diseases, or vasculitis. Blood glucose level, thyroid function, and HIV serology were normal in all patients. One patient had mild chronic hepatitis C (Case 17) but no serology for other viral hepatitis. CSF analysis of protein, glucose, cell count, viral PCR and bacterial growth were normal in all 14 patients studied. In a patient with CVST, CSF studies revealed pleocytosis. No markers for abnormal coagulopathy were detected in other patients.

### Neuroimaging findings

#### Classical PRES (cases 2–7)

**Fig. 1:**
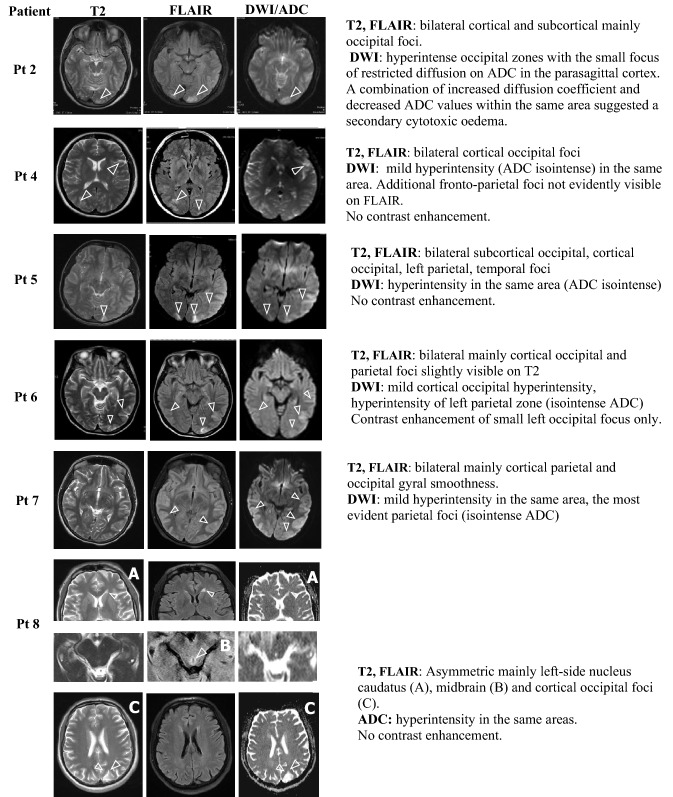
PRES in cases 2, 4–8

Focal lesions in the brain MRI or CT were detected within 24 h of AE with seizures, blurred vision, or confusion (Fig. 1, Table 3). The lesions were asymmetrically bilateral, cortical, or subcortical multifocal, and either frontotemporal or parieto-occipital, or both, representing PRES. Frontotemporal lesions were associated with seizures and blurred vision with occipital lesions. The size and shape of the lesions varied.

The lesions were hyperintense in diffusion-weighted (DW) MRI in all cases, and either hyperintense or isointense in Apparent Diffusion Coefficient (ADC) suggesting vasogenic oedema. In Case 2, the small focus of decreased ADC values suggested secondary cytotoxic oedema (Fig. 1). Contrast enhancement was usually unremarkable. Phase contrast MR-angiography was normal in four out of five patients studied, including Case 2 with severe headache, which excluded reversible vasoconstriction syndrome (RVCS). Reversible segmental vasoconstriction was detected in Case 1 with concomitant CVST (Fig. 3G and Table 3). In Case 3, PRES was detected on brain CT, which revealed symmetric hypodense foci (20 and 21 HU) in the subcortical white matter of the frontotemporal lobes, 13 and 22 mm.

#### Basal ganglia and brain stem oedema (case 8)

Case 8 with a short-lasting delirium demonstrated nucleus caudatus and midbrain hyperintensity comparable with Wernicke encephalopathy (Fig. 1A, B) and occipital cortical oedema (Fig. 1C). This could be due to the single alcohol binge followed by acute pancreatitis three weeks prior to the manifestations of the attack with confusion, severe abdominal pain and PNP. Porphyrinogenic medication and rapid weight loss before confusion triggered the attack. Hyponatremia (125 mmol/L) was corrected slowly without signs of pontine myelinolysis. The patient was the only case with both normal serum ALT (< 50 U/L) and PRES. Serum vitamin B1-level was normal at the time of imaging.

#### PRES and sinus thrombosis (case 1)

**Fig. 2:**
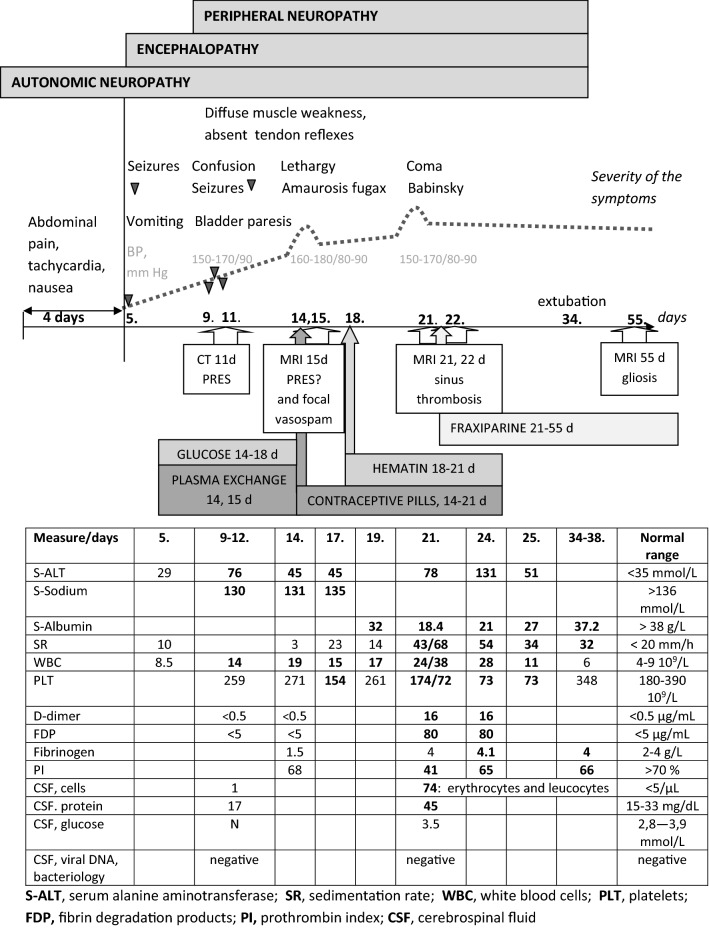
The natural course of an acute attack complicated by cerebral venous sinus thrombosis (CVST) in case 1

**Fig. 3:**
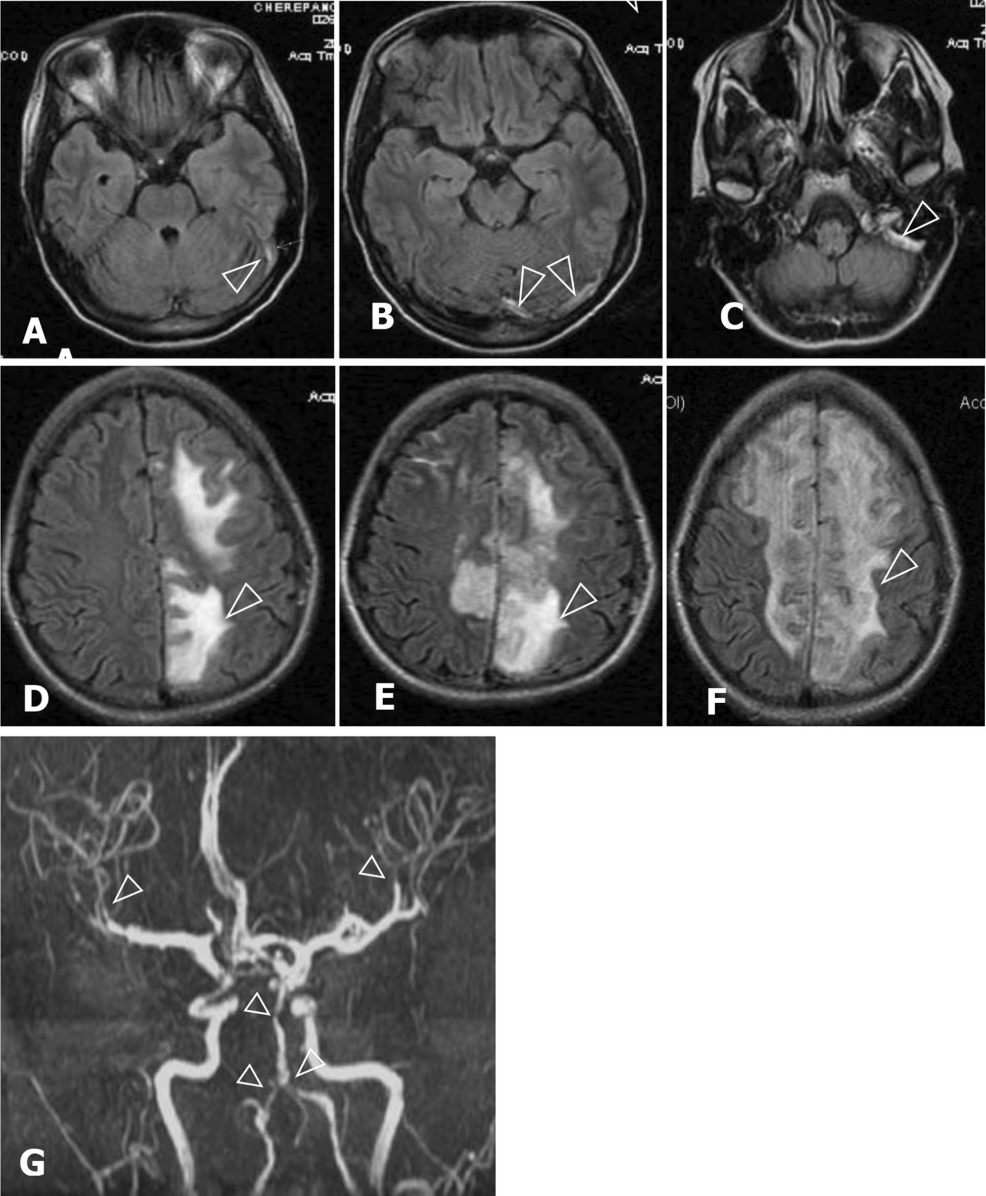
Cerebral venous sinus thrombosis (left transverse and sigmoid sinuses) in case 1. **A**–**C** FLAIR: areas of the increased signal intensity at the sinus location at 10. day of encephalopathy. **D**, **E** FLAIR: progression of focal lesions within 1.5 months (**D** 10. day, **E** 17. day, **F** 50. day of encephalopathy) leading to diffuse gliosis. **G** 3D-time of flight (TOF) MR angiography: vascular narrowing and uneven shapes of basilar and both vertebral arteries suggesting segmental vasospasm at the 10 day

The most prominent finding was CVST on the brain MRI of Case 1 (Tables 2, 3, Figs. 2 and 3). A 26-year-old female had experienced recurrent undiagnosed attacks during premenstrum. At the onset of an attack, she had severe abdominal pain, tachycardia, and systolic hypertension (150–170/90 mmHg) without headache. During the following four days, the attack proceeded to seizures, progressive confusion and later generalised muscle weakness with low tendon reflexes due to PNP (Fig. 2). Brain CT during the period of seizures (11th day of the attack) revealed hypodense foci in the left frontal and parietal area. EEG revealed focal epileptiform discharges in the frontal area. She was diagnosed with AHP and treated with 10–20% glucose infusions before hematin was available. Plasmapheresis, which has been used experimentally during attacks without clinical effect, did not improve her condition either. Contraceptive pills were administered when she was recovering from the attack to prevent further cyclical attacks (Fig. 2). Thereafter, she became lethargic and developed short-lasting blurred vision and reversible motor dysphasia. Brain MRI (15th day of the attack) revealed hyperintensive foci with the same distribution as in the CT a week earlier suggesting PRES. Segmental vasospasms of the basilar, both vertebral arteries and M2–M4 segments of the middle cerebellar arteries could be detected. She was treated with the hematin infusions (Normosang, Recordati Rare Diseases, France) for four days from the 18th day of the attack.

The patient’s condition deteriorated to coma during the following week. The previous brain MRI was re-evaluated, and transverse sinus thrombosis could be identified retrospectively (Fig. 3). Brain MRI (21st day of acute attack) revealed progressing CVST. Control brain MRI (22nd day of the attack) revealed haemorrhagic focus in the left parietal area and progressing oedema.

Elevated sedimentation rate, d-dimer, fibrinogen, fibrin degradation products (FDP), and transient thrombocytopenia were concomitant with the diagnosis of CVST. Coagulopathy was evident only after the second week (Fig. 2). The patient had no signs of thrombophilia: protein C and S, and anti-thrombin levels were normal; Factor V mutation analysis, antinuclear and anti-cardiolipin antibodies were negative, but several thrombogenic factors such as severe acute illness, hyponatremia, hypoalbuminemia, and immobilisation were present. No signs of infection or markers of autoimmune disorders were present. Oestrogen containing contraception pills, which should be avoided during an attack, and hematin treatment could have contributed to the progression of thrombosis. She was treated with nadroparin subcutaneously, which resulted in a partial recovery.

Hereditary coproporphyria was confirmed by plasma porphyric emission spectrum (positive at 619 nm), 20-fold increases in urinary and faecal excretion of coproporphyrin (isomers I < III) and a mutation in the coproporphyrinogen oxidase gene (p.L94P).

Control brain MRI (55th day of the attack) revealed profuse gliosis in both frontal and parietal areas (Fig. 3) and brain MR angiography was normal. In the follow-up of 2 years, clinical sequelae such as mild cognitive decline with the frontal deficiency and asymmetric left-sided central tetraparesis remained as the signs of chronic encephalopathy**.**

#### SIADH (case 9)

**Fig. 4:**
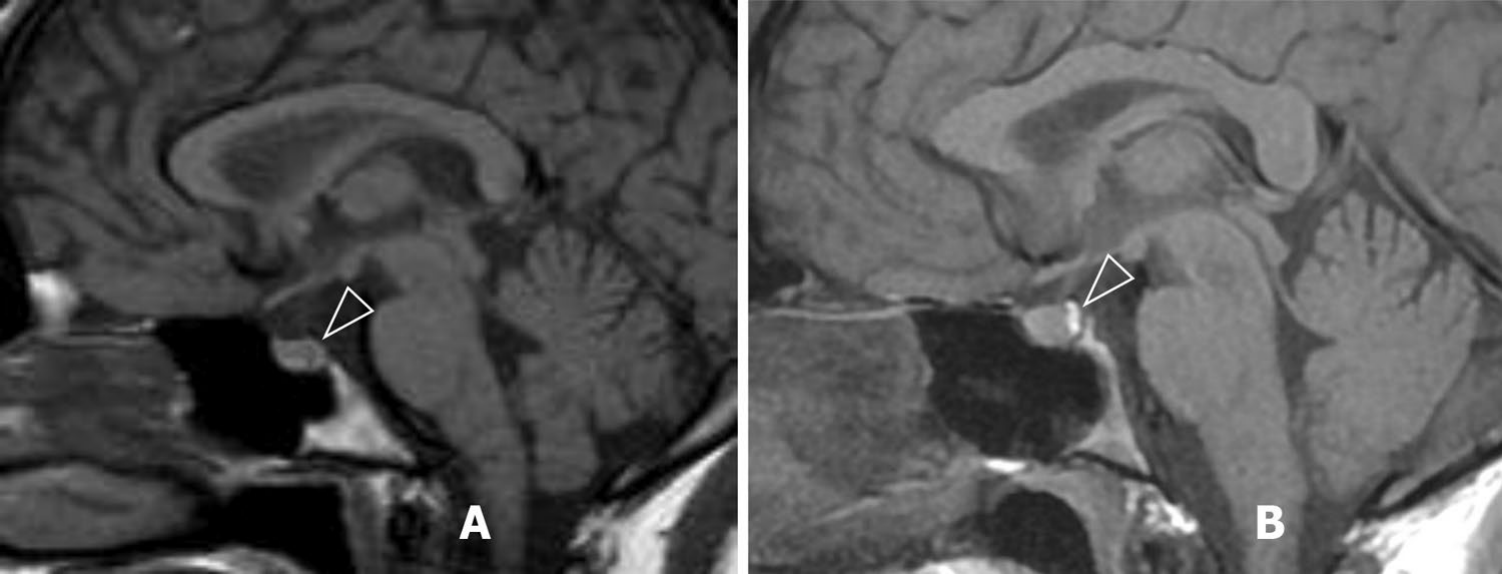
T1-weighted MR imaging revealed a reduced bright signal from the posterior pituitary gland in case 9 (**A**, arrowhead) in comparison to the normal bright signal in case 4 (**B**, arrowhead)

Her brain MRI showed no signs of PRES despite the clinical manifestations of AE with confusion and Babinski signs accompanied by abdominal pain and rhabdomyolysis (CK 21,806 U/L, normal range < 150 U/L) during neuroimaging (Fig. 4).

The patient had severe hyponatremia (Na 108 mmol/L, normal range 135–145 mmol/L) due to SIADH nine days prior to MRI [31]. Even though the plasma sodium level had normalised before neuroimaging, a reduced signal of ADH-containing neurohypophysis at T1 images in the posterior pituitary was present. The patient recovered from a severe attack after nine days without sequelae.

#### Acute encephalopathy and normal brain MRI (cases 10–19)

In about half of the cases neuroimaging was unremarkable, despite severe AE manifested with confusion and/or hallucinations. Lower sensitivity of CT, incorrect MRI timing several days after the peak of AE could partly explain this (Table 3). Brain MRI performed just 12 h after the episode of generalised seizures and 3-h postictal confusion was unremarkable indicating that even short-lasting encephalopathy with seizures may not be enough to produce PRES-like foci (Case 13, Table 3).

### Prognosis of porphyric encephalopathy

Most patients (89%) recovered without clinical and radiological sequelae. The patients without co-existing PNP were discharged 10–15 days later without clinical sequelae, even though residual lesions of PRES were still present in three cases. Brain MRI became normal 1.5 months later in two cases, and only one patient had minimal residual foci.

Poor prognosis of AE was caused either by a diagnostic delay > 1 month or concomitant CVST. In contrast to PNP with a prolonged recovery, AE was quickly reversible. One patient died of an attack due to ventilator-associated pneumonia, complicated by septicemia and coagulopathy. Brain autopsy demonstrated grossly normal brain tissue similar to reported previously [4, 6].

### PEPT2 polymorphism

The most common haplotypes in our cohort were PEPT2*1/1 (corresponding to the genotype exon 13C/exon 15C; 38%, 21 subjects) and PEPT2*1/2 (exon 13C/exon 15T; 39%, 22 subjects). PEPT2*2/2 haplotype (exon 13T/exon 15T) was detected among 10 subjects (18%) and other PEPT2 variants of unknown significance among three subjects (5%). There was no correlation between PEPT2 variants and AHP subtypes (*P* = 0.956). Furthermore, the PEPT2 haplotype did not correlate with AHP phenotype (*P* = 0.363).

PEPT2 haplotype showed no correlation with the prevalence of AE (two-sided *P* < 0.05 for all). In contrast, PEPT2*2/2 haplotype was more common among patients without encephalopathy than with encephalopathy (25.8% vs. 8.3%, respectively, *P* = 0.159). Similarly, no correlation was detected between the PEPT2 haplotypes and polyneuropathy (two-sided *P* < 0.05 for all). Of note, PEPT2*1/2 haplotype was commoner among patients with polyneuropathy than without (52.4% vs. 31.4%, respectively, *P* = 0.161), and PEPT2*2/2 was less common (9.5% vs. 22.9%, respectively, *P* = 0.207).

Chronic kidney disease was diagnosed among nine patients of the 48 patients (19%) with sufficient data available. Chronic kidney disease was most common among patients with PEPT2*2/2 haplotype, although this did not reach statistical significance (*P* = 0.192).

## Discussion

### Clinical and radiological findings

In this study, a triad of seizures, confusion, and blurred vision was the most common clinical pattern of AE during an acute attack. Since severe encephalopathy is a rare complication of an attack, other aetiologies should be excluded promptly using neuroimaging.

#### PRES

Reversible multifocal lesions corresponding to PRES were detected in 42% of our 19 patients with severe AE and AHP. In another series including eight patients with AE, only one patient (12.5%) had PRES [32].

Radiological picture of PRES in AHP is similar with PRES of other origin [33]. PRES is an unspecific radiological finding resulting from reversible vasogenic oedema [33] supported by increased both DW and ADC values in the same lesions [4]. The small focus of cytotoxic oedema with restricted diffusion verified by decreased ADC in our Case 2 has been described in few cases with AE and PRES [5, 34]. Cytotoxic oedema results from longer-lasting oedema than those of a purely vasogenic origin. No DW restriction correlates with quicker radiological recovery.

A combination of classic cortical–subcortical PRES with brainstem and basal ganglia involvement in Case 8 has rarely been described in PRES [35]. Few similar cases of AHP with deep brain foci have been published [5, 36, 37]. Alcohol consumption and malnutrition could have been additional triggering factors of AE in this case with PRES and Wernicke-like encephalopathy [38].

#### MR angiography

In our series including five patients studied by MRA, transient segmental vasoconstriction could be detected only in Case 1 with PRES and CVST. In other series including 12 patients with AHP and PRES studied by MRA or conventional angiography, RVCS and PRES have been identified simultaneously in five cases [29]. These patients were more severely affected and resulted in radiological and clinical sequelae. The similar frequency of RVCS has been reported in PRES of other origin [33, 39].

*MR spectroscopy* performed in two cases with AHP and PRES was unremarkable [40, 41], excluding lactate overload and deficiency of haem-containing cytochrome oxidases as seen in mitochondrial encephalomyopathy, lactic acidosis and stroke-like episodes (MELAS) [42].

#### Radiological evidence of SIADH

The absence of hypophyseal ADH-bright signal after severe hyponatremia in Case 9 supports the primary role of increased membrane permeability and the leakage of ADH from the neurohypophysis storage in the development of SIADH in AHP.

#### PRES and CVST

Cerebral venous thrombosis has not been previously reported in AHP. The combination of CVST and PRES has been reported only in a few patients with eclampsia [43], or in haematological oncology [44]. Even though endothelial dysfunction is a key factor in the pathogenesis of both conditions, the pathogenesis of CVST and PRES differ substantially. CVST in Case 1 occurred most likely due to secondary causes such as plasmapheresis-induced hypoalbuminemia, and activation of the coagulation pathway by contraceptive pills and heme infusions, dehydration and anaemia [45]. Since hormonal preparations such as contraceptive pills can be both porphyrinogenic and thrombogenic, they should not be administered to patients during an attack. PRES could have been a predisposing factor to CVST due to increased intracerebral pressure and venous stasis [44].

The clinical pictures of PRES and CVST overlap and can only be distinguished by neuroimaging. It is crucial to recognise CVST promptly and prevent secondary complications such as brain infarctions, intracranial haemorrhage and severe neurological sequelae [44].

#### PRES due to AHP vs. eclampsia

Hormonal and weight changes after delivery can be triggering factors for an attack, as in Cases 11 and 13. The clinical and radiological picture of encephalopathy partly coincides in both acute attacks and eclampsia [29, 46]. In contrast, a combination of dysautonomia, PNP and hyponatremia in the absence of proteinuria are atypical for eclampsia [46].

#### Clinical manifestations of porphyric encephalopathy with PRES

PRES represents the most severe type of AE, since seizures, confusion, hallucinations and focal CNS signs are more frequent among patients with PRES during an attack than in any of the previously published series (Table 1) [29].

The clinical picture of PRES-associated encephalopathy in AHP is similar with PRES of other origin, except for the rarity of headaches (25% in our series, 13% in previously published *vs.* 50% in PRES of other origin) [29, 33]. This could be due to the decreased level of consciousness and abdominal pain exceeding the severity of headache in some cases. The patients with AE and severe headache during an attack do not necessarily complain of abdominal pain or manifest PNP (Cases 10, 14, 16), which suggests different mechanisms of encephalopathy (endothelial dysfunction, permeability failure), and autonomic and polyneuropathy (direct neurotoxicity of porphyrin precursors). Seizures have been reported in 85% of the published cases with acute porphyria and PRES (*n* = 46) [29] and in 63% in our series (Table 3). Seizures reflect the severity of encephalopathy [47] and could be secondary to PRES but can also deteriorate it. Seizures should be treated immediately with non-porphyrinogenic anticonvulsants, such as levetiracetam [48] or benzodiazepines, but the patients do not need anti-epileptic therapy after recovery from the attack [30].

### Pathogenesis of PRES in acute porphyria

Triggering factors of PRES reported from several diseases with multiple pathogenesis include severe hypertension, anti-VEGF cytotoxic drugs, eclampsia and autoimmune diseases leading to transient endothelial dysfunction [33, 39]. Of the metabolic diseases, PRES has been reported in renal and hepatic failure, and severe electrolyte imbalances including hypercalcemia, hyper- and hyponatremia in addition to AHP [33, 49]. The common features in these conditions include moderately elevated BP and an acute nature of metabolic disturbances otherwise different from AHP.

Since PRES could be very mild resolving within a few hours [33], it is difficult to predict, if vasogenic oedema is always present in porphyric encephalopathy. However, it seems that oedema is a universal mechanism of severe encephalopathy in AHP, and it is just a matter of the technical sensitivity to detect it.

The pathophysiology of BBB disruption has not been established in AHP or in PRES of other origin [29, 33]. The main hypotheses are (1) disruption of the vessel reactivity via rapid increase of blood pressure or vasoactive metabolites such as NO or CO; (2) direct toxicity to endothelial cells of BBB via pro-inflammatory cytokines or chemokines [29, 33, 39]; (3) abnormal functioning of aquaporins (AQPs), astrocyte water channels [29, 33, 39].

Currently, ALA is the only metabolite shown to be neurotoxic for autonomic and peripheral nerves during an attack (Table 4) [15]. ALA has very low BBB permeability leading to low concentration in CSF, insufficient to cause neurotoxic effect [15–17]. In contrast, the endothelial dysfunction resulting in permeability failure, but not direct neurotoxicity of ALA, could be a key element in the pathogenesis of PRES-associated encephalopathy in AHP similarly to many other PRES [29]. The exclusive factors causing endothelial dysfunction in AHP has not yet been established (Table 4).

**Table 4:** Main hypotheses of PRES pathogenesis in AHP

Main hypotheses	Evidence in vitro or in vivo	Limitations	Potential role in PRES of AHP
*I. Specific for acute porphyria*			
A. ALA excess obligatory during an attack	Direct neurotoxicity in vitro	Low permeability for BBB, not neurotoxic at concentrations detected in CSF	Could not explain PRES
	Structural similarity to GABA, antagonism to receptors in vitro at lower concentrations	Could explain milder mental symptoms but not PRES	Could not explain PRES
	ROS excess due to ALA auto-enolisation in vitro	Demonstrated at the ALA concentrations 100-fold exceeded the levels during acute attacks	Could not explain PRES. Only minor input in pathogenesis of encephalopathy possible
B. Heme deficiency: *NOS and mitochondrial oxidative phosphorylases*	Treatment with hematin quickly resolves autonomic neuropathy and encephalopathy Mild heme deficiency was shown in the liver enzymes but controversial results in muscles and brain	Exogenous heme does not cross BBB Heme deficiency in CNS has not been proven: NOS activity in HMBS-/- mice brains was normal S-NOS concentration in a patient with PRES was normal mean BP < 160 mm Hg MR spectroscopy did not reveal lactic peaks in 2 patients with AHP and PRES	No evidence of deficient NOS No evidence of abnormal mitochondrial functioning in brain, no similarity with MELAS
C. Autonomic neuropathy	100% in any acute attack	Humoral myogenic autoregulation prevails of neurogenic in the brain at mean BP < 160 mm Hg	Could serve as a co-factor decreasing the upper limits of autoregulation
D. PEPT 2 polymorphisms	Play a role for kidney damage in AHP and for neurotoxicity in chronic lead intoxication by decreasing ALA efflux from CSF	No correlation in our study	Needs more data
*II. Unspecific for porphyria, suggested in PRES of other origin*			
E. Hypertension	70% in PRES of AHP (Jaramillo-Calle et al. [29])	Usually not severe (mean BP > 160 mm Hg), but abrupt	Could serve as co-factor for PRES
F. Hyponatremia	55% in PRES of AHP (Jaramillo-Calle et al. [29])	Usually not severe and not obligatory	Could serve as co-factor for PRES
G. SIADH	The main cause of hyponatremia in AHP	SIADH per se may be secondary to the increased membrane permeability	Could serve as co-factor for PRES
H. Elevated cytokines of hepatic origin	Cytokines, chemokines and growth factors were elevated in AIP patients in remission vs. healthy controls in one study Elevated S-ALT in patients with PRES vs. normal values in patients without encephalopathy during acute attacks	Never tested in acute attacks vs. remission No signs of inflammation in routine laboratory studies	Could explain PRES since acute phase proinflammatory cytokines are produced in the liver, but no evidence during an acute attack so far

#### Dysregulated vascular tone of cerebral arteries

Several lines of evidence support increased vascular reactivity in different organs during acute attacks in patients with AHP and HMBS − / − mice, e.g. kidney, retina and mesenteric arteries [50]. Reversible cerebral vasoconstriction has been detected in half of the cases with PRES studied by angiography [29].

Acute autonomic neuropathy*,* which manifests mainly as cholinergic deficiency with secondary sympathetic activation, is present in 100% of attacks [1]. This could be one explanation of abundant vasoconstriction or vasodilation during an attack, since dysautonomia has directly affected the endothelial function in animal models [51]. If cerebral blood pressure does not exceed critical values (mean 60–160 mmHg), humoral myogenic autoregulation mechanisms prevail over neurogenic regulation. Thus, autonomic dysfunction alone cannot explain disrupted vascular tone in the brain, but could serve as a co-factor decreasing the upper limits of autoregulation [51].

*Hypertension* is a common sign of an attack due to autonomic neuropathy. It is present in 70% of the cases with PRES due to AHP, but hypertensive crisis is infrequent [29]. Malignant hypertension was the first factor reported to cause PRES alone via disrupted autoregulation of cerebral flow [52]. Moreover, mild hypertension is commonly detected in PRES of other origin [33]. Although blood pressure was only moderately elevated during an attack in our series, the rise can still be dramatic in most patients, who are mainly young females with low normal blood pressure in remission. Hypertension unlikely explains PRES in AHP, but can be an important co-factor in cases with pronounced fluctuations of blood pressure [33].

#### Haem deficiency in the brain

Mild haem protein deficiency during an attack has been demonstrated in the liver [15, 19]. Significant alteration of NO level in addition to endothelin-1, has been demonstrated in symptomatic AHP patients, but the origin of this alteration is unclear [53]. The deficiency of haem-containing endothelial nitric oxide synthase (NOS) 1 in the brain has been assumed to be responsible for NO deficiency and cerebral vasoconstriction [54]. The activity and mRNA expression of NOS1 have been normal in the brain homogenates of the HMBS (−/−) mice after induction of ALAS with phenobarbital [19]. Moreover, serum NOS concentration was normal in the AIP patient during PRES accompanied by RVCS [34]. Absence of lactate peaks typical of mitochondrial deficiency in MR spectroscopy in two cases with AHP and PRES excludes deficiency of cerebral oxidative phosphorylases [18, 34, 40, 42].

Exogenous haem does not cross BBB, but alleviates AE by correcting the liver metabolism, and liver transplantation brings an immediate cure for patients with severe recurrent attacks [55]. Thus, the liver is the most likely source of endothelial toxins in AHP.

#### Potential factors of abnormal BBB permeability

*Hepatic cytokines*. A high level of porphyrin metabolites could result in acute toxic and/or inflammatory hepatitis [56]. Interestingly, our patients with AE had acute 1.5–5-fold increases in liver transaminases in contrast to normal values during typical attacks with dysautonomia and pain solely.

Pro-inflammatory factors such as IL-6 or cytokines, which originate from the liver during an attack, could be a reasonable explanation for PRES in AHP, similar to other encephalopathies such as eclampsia, autoimmune diseases or cytotoxic medications [29, 33]. The main limitations of this hypothesis include the absence of relevant systemic inflammation sufficient to cause endothelial toxicity during an attack when assessed by inflammatory or autoimmune responses in our and previously published cases of AE [4, 37, 40, 41, 57–59]. A single study has revealed mild elevation of cytokines, chemokines and growth factors in symptomatic patients with AIP in remission, suggesting mild inflammation even in remission [60].

Elevated levels of ROS originating from auto-enolisation of ALA have been detected in experimental studies of AHP [61]. This effect is present only under very high concentrations of ALA, which have not been detected during acute attacks, and thus, the role of ROS in PRES is most likely limited [62].

Hyponatremia is common, present in 88% of acute attacks with PRES in our series and in 55% of previously published [29], which is significantly more common than otherwise in PRES [33]. Hyponatremia in AHP can be multifactorial, but it is mainly due to SIADH during AE. Hyponatremia could mediate PRES either directly by increased BBB permeability and vascular reactivity due to osmotic factors, or indirectly by elevated ADH via increased secretion of VEGF, accelerated transcription of aquaporin 4 in astrocytes, or activation of vasopressin V1a receptors disturbing cerebral vascular reactivity [63–65]. SIADH seems to be a secondary phenomenon of the membrane leakage based on MRI findings in Case 9 but has a significant role in the cascade of factors increasing BBB permeability in AHP.

Nausea induces ADH excretion dramatically via neural stimulus and rigorous fluid therapy may aggravate hyponatremia. Clinicians should be careful with fluid therapy not to cause potential acute renal impairment with too strict volume restrictions or too rigorous fluid therapy to aggravate hyponatremia causing CNS complications.

#### PEPT2 polymorphisms

Only 1% of ALA crosses the unaffected BBB, mainly taken up by PEPT2 in choroid plexuses [15–17, 20]. Theoretically, the patients with the less active form of PEPT2*2/2 should have higher susceptibility than PEPT2*1/2 to porphyric encephalopathy by decreased ALA clearance from the CSF [23]. The same polymorphism brings lower susceptibility to nephropathy by decreased tubular re-uptake of ALA [27]. According to this theory, patients with AHP are either predisposed to encephalopathy or nephropathy. In contrast, the PEPT2*2/2 haplotype was less common among patients with encephalopathy and/or polyneuropathy compared to PEPT2*1/2 haplotype in our series, but more common in patients with chronic kidney disease. This did not reach statistical significance probably due to the small number of patients. Our results demonstrate that PEPT2 haplotypes most likely have only a minor role, if any, in developing AE.

#### The pathogenesis of mild porphyric encephalopathy without PRES

In addition to the direct neurotoxicity of ALA suggested for autonomic and peripheral nerves, concurrent agonism of GABA receptors in the brain tissues has been demonstrated in animal models even at low concentrations of ALA [15, 66]. This could explain mental symptoms during an attack without producing radiologically evident lesions. Modulating CNS signal transduction by ALA could be involved in patients with milder symptoms of porphyric encephalopathy, suggesting two different pathogenetic entities of porphyric encephalopathy: GABA-mediated metabolic encephalopathy and PRES.

## Conclusions

1. Acute encephalopathy in AHP manifests with a combination of mental symptoms and confusion, seizures, and SIADH, but rarely with focal CNS deficits, except for blurred vision. It is caused by reversible, multifocal brain oedema visualised as PRES on brain MRI.
2. The clinical picture of PRES-associated encephalopathy in AHP is similar with PRES of other origin, except for the rarity of headache.
3. Findings of PRES in patients with AE should alert radiologists and neurologists to look for acute porphyria, especially if patients are young women. In addition, venous sinus thrombosis should be excluded.
4. Mild encephalopathy at the early phase of an acute attack possibly caused by ALA-promoted modulation of neural GABA/glutamate transduction and severe encephalopathy with PRES are two different pathogenetic entities.
5. The pathogenesis of PRES in AHP is multifactorial and still unclear. Acute endothelial dysfunction resulting in permeability failure could be explained by a combination of abrupt hypertension and increased vascular reactivity as signs of autonomic neuropathy induced by metabolic and inflammatory factors of hepatic origin. PRES can be accelerated by SIADH and vice versa, and it may aggravate SIADH by increasing the BBB permeability also in the neuro-hypophyseal area.
6. The role of haem deficiency in neural tissues is still controversial. Even though urine and plasma levels of PBG and ALA are commonly extremely high during AE reflecting the biochemical severity of an acute attack, the neurotoxicity of ALA cannot explain PRES due to very low BBB permeability leading to low concentration in CSF, insufficient to cause neurotoxic effect.
7. Our results demonstrate that PEPT2 haplotypes are unlikely to contribute to pathogenesis of AE.
